# Microbial aetiology of acute diarrhoea in children under five years of age in Khartoum, Sudan

**DOI:** 10.1099/jmm.0.000043

**Published:** 2015-04

**Authors:** Amir Saeed, Hadi Abd, Gunnar Sandstrom

**Affiliations:** Karolinska Institute, Department of Laboratory Medicine, Division of Clinical Microbiology F 68 and Karolinska University Hospital, Huddinge, SE-141 86 Stockholm, Sweden

## Abstract

Diarrhoea is one of leading causes of morbidity and mortality worldwide. Recent estimations suggested the number of deaths is close to 2.5 million. This study examined the causative agents of diarrhoea in children under 5 years of age in suburban areas of Khartoum, Sudan. A total of 437 stool samples obtained from children with diarrhoea were examined by culture and PCR for bacteria, by microscopy and PCR for parasites and by immunoassay for detection of rotavirus A. Of the 437 samples analysed, 211 (48 %) tested positive for diarrhoeagenic *Escherichia coli*, 96 (22 %) for rotavirus A, 36 (8 %) for *Shigella* spp., 17 (4 %) for *Salmonella* spp., 8 (2 %) for *Campylobacter* spp., 47 (11 %) for *Giardia intestinalis* and 22 (5 %) for *Entamoeba histolytica*. All isolates of *E. coli* (211, 100 %) and *Salmonella* (17, 100 %), and 30 (83 %) isolates of *Shigella* were sensitive to chloramphenicol; 17 (100 %) isolates of *Salmonella*, 200 (94 %) isolates of *E. coli* and (78 %) 28 isolates of *Shigella* spp. were sensitive to gentamicin. In contrast, resistance to ampicillin was demonstrated in 100 (47 %) isolates of *E. coli* and 16 (44 %) isolates of *Shigella* spp. In conclusion, *E. coli* proved to be the main cause of diarrhoea in young children in this study, followed by rotavirus A and protozoa. Determination of diarrhoea aetiology and antibiotic susceptibility patterns of diarrhoeal pathogens and improved hygiene are important for clinical management and controlled strategic planning to reduce the burden of infection.

## Introduction

Diarrhoeal diseases remain one of the leading causes of preventable death in developing countries, especially among children under 5 years of age. In 2008 alone, around nine million children under 5 years died and around 40 % of those deaths were due to pneumonia and diarrhoea (You *et al.*, 2010). Globally, diarrhoea is the second largest cause of death in children under 5 years of age, causing one in every five deaths. Unfortunately, diarrhoea kills more children than AIDS, malaria and measles combined (WHO, 2008).

Diarrhoea is common in the developing countries, especially in areas with poor hygiene and sanitation and with limited access to safe water. Other conditions, such as malnutrition, may further increase the risk of contracting diarrhoea in developing countries. These factors may lead to a significant disease burden and negative economic effects, resulting from medical costs, loss of work, lower quality of life and high mortality.

Infectious organisms, including bacteria, viruses, protozoa and helminths, cause diarrhoea. These organisms are transmitted from the stool of one individual to the mouth of another, a route termed faecal–oral transmission. However, they differ in the exact route of entry from stool to mouth and in the infectious dose needed to cause the illness. *Escherichia coli* is considered to be the aetiological agent of many diseases, including some affecting the urinary tract and intestine. The classification of diarrhoeagenic *E. coli* (DEC) strains is based on their virulence properties and comprises six groups: enterotoxigenic *E. coli* (ETEC), enteropathogenic *E. coli* (EPEC), enteroinvasive *E. coli* (EIEC), enterohaemorrhagic *E. coli* (EHEC), enteroaggregative *E. coli* (EAggEC) and diffuse adhering *E. coli* (DAEC) (Nataro & Kaper, 1998).

In Sudan, diarrhoea is one of the most common reasons for children to visit healthcare clinics, but knowledge of the causative agents of these diarrhoea cases is limited.

The enteric pathogens rotavirus and DEC are the most common causes of diarrhoea globally (Parashar *et al.*, 1998) with DEC cited as the most important cause in developing countries (Gomes *et al.*, 1991). Rotavirus is the leading cause of acute infantile gastroenteritis globally and is responsible for 20 % of deaths in children under 5 years of age (de Zoysa & Feachem, 1985).

In addition to rotavirus and DEC, other enteropathogens including *Shigella* spp., *Salmonella* spp., *Vibrio cholerae* and *Campylobacter* spp. may cause diarrhoea.

In Sudan infant mortality is 102 per 1000 live births and neonatal mortality is 51 per 1000 live births. Khartoum is the capital city of Sudan with a total population of 5 million. It is located in the centre of Sudan and administratively divided into seven localities. Around 80 % of the population of Khartoum State population live in the urban area (El Tayeb *et al*., 2014).

Infectious diseases cause most of these deaths (UNICEF, 2014), but the aetiological agents are usually not known and therefore misuse of antibiotics is common. This has led to antibiotic resistance becoming a major problem in Sudan.

The aims of this study were therefore to identify the aetiology of diarrhoea in Khartoum, Sudan, and to determine the antibiotic susceptibility of the organisms isolated.

## Methods

### 

#### Sample collection.

Faecal samples (one per subject) were collected in sterile containers from children with diarrhoea (defecation more than three times within 24 h of hospital admission) in the period from January 2013 to December 2013 . All faeces were stored in the collection containers with Cary–Blair transport medium at 4 °C and transported to the microbiological laboratory within 24 h. The residue of each sample after the first culture on medium was kept at −70 °C for further analyses.

#### Isolation and identification of diarrhoeal pathogens.

Stool samples were processed and analysed for micro-organisms and intestinal parasites at the laboratory of the University of Medical Sciences and Technology Hospital (UMST) (Khartoum, Sudan). Standard culture and identification methods were used to identify enteric pathogens (WHO, 1987). Intestinal parasites were identified by direct microscopy of wet mount preparations. Positive samples for *Entamoeba* were identified to species level by PCR to differentiate between *E. histolytica* and *E. dispar* (Saeed *et al.*, 2011). For group A rotavirus, stool samples were analysed using the SD BIOLINE Rota Rapid test (catalogue no. 14 FK10; Standard Diagnostics) as described by the manufacturer.

For DEC, *Shigella* spp. and *Salmonella* spp., stool samples were cultured on MacConkey agar, thiosulphate citrate bile salt (TCBS) and deoxycholate citrate agar (for *Shigella* spp. and *Salmonella* spp.). All plates were incubated overnight at 37 °C. All samples were tested for *Vibrio cholerae*, *Shigella* spp. and *Salmonella* spp. by colony morphology, biochemical properties and agglutination with specific antisera (Mast Diagnostica). For *Campylobacter* spp., the stool samples were inoculated on blood-free charcoal-based selective medium (CCDA) (bioMérieux) and blood-containing medium selective for *Campylobacter* spp. (bioMérieux). A multiplex PCR using eight primer pairs specific for the virulent genes of five different types of DEC was used for the identification of *E. coli* from the subcultured bacteria (Table 1) (Nguyen *et al.*, 2005).

**Table 1.** Primers used for multiplex PCR for detection of diarrhoeagenic *E. coli*

Primer	Target gene	Primer sequence	bp
LT	*eltB*	5′TCTCTATGTGCATACGGAGC3′	322
5′CCATACTGATTGCCGCAAT3′
ST	*estA*	5′GCTAAACCAGTAGAGGTCTTCAAAA3′	147
5′CCCGGTACAGAGCAGGATTACAACA3′
VT1	*vt1*	5′GAAGAGTCCGTGGGATTACG3′	130
5′AGCGATGCAGCTATTAATAA3′
VT2	*vt2*	5′ACCGTTTTTCAGATTTTGACACATA3′	298
5′TACACAGGAGCAGTTTCAGACAGT3′
eae	*eaeA*	5′CACACGAATAAACTGACTAAAATG3′	376
5′AAAAACGCTGACCCGCACCTAAAT3′
SHIG	*ial*	5′CTGGTAGGTATGGTGAGG3′	320
5′CCAGGCCAACAATTATTTCC3′
bfpA	*bfpA*	5′TTCTTGGTGCTTGCGTGTCTTTT3′	367
5′TTTTGTTTGTTGTATCTTTGTAA3′
EA	*pCVD*	5′CTGGCGAAAGACTGTATCAT3′	630
5′CAATGTATAGAAATCCGCTGTT3′

#### Antimicrobial sensitivity.

The antibiotic sensitivity of the micro-organisms isolated from the faeces of children to different antibiotics was tested using Mueller–Hinton agar (Himedia) and the standard disc diffusion technique of the modified Kirby–Bauer method, as recommended by the Clinical and Laboratory Standards Institute (CLSI). For the disc susceptibility testing (BD), disc content and zone size scoring were as follows; ampicillin (10 mg) ≥17 mm sensitive and ≤13 mm resistant, amikacin (30 µg) ≥17 mm sensitive and ≤14 mm resistant, ceftazidime (30 µg) ≥18 mm sensitive and ≤14 mm resistant, gentamicin (10 µg) ≥15 mm sensitive and ≤12 mm resistant and nalidixic acid (30 µg) ≥19 mm sensitive and ≤13 mm resistant were used.

#### Statistical analysis.

The chi-squared (χ^2^) test was used to determine the statistical significance of the data. A *P*-value of <0.05 was considered statistically significant.

## Results

### Occurrence of pathogens

**Fig. 1.**
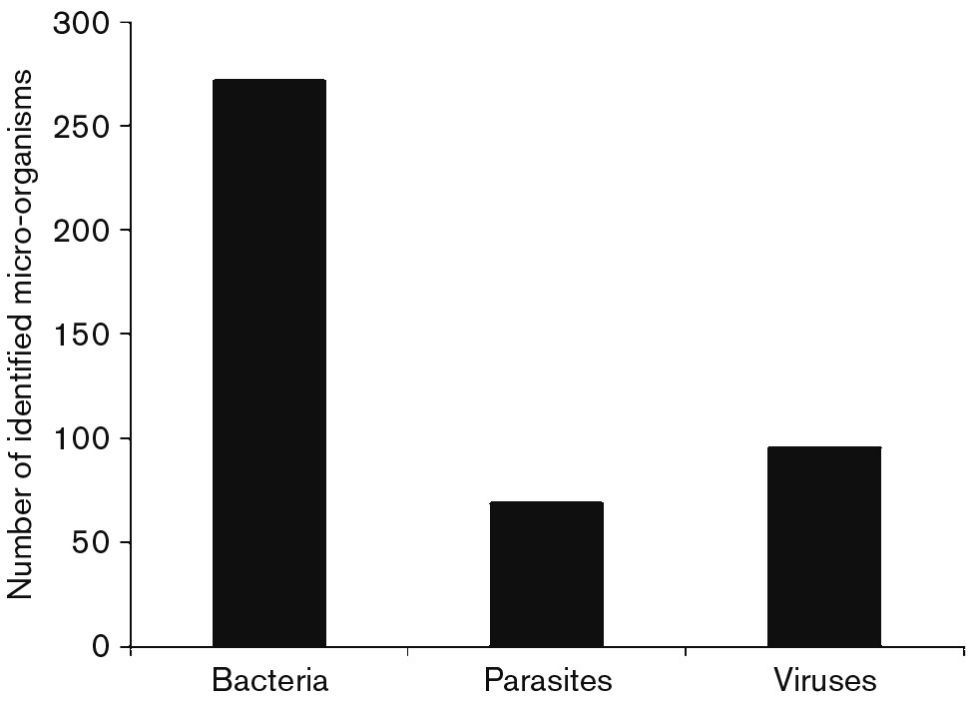
Total number of different aetiological agents found in the 437 diarrhoeal cases included in the study.

Bacterial infection was the main cause of diarrhoea in children, and among the total cases of bacterial infection (Fig. 1), *E. coli* caused 48 %, *Shigella* spp. caused 8 %, *Salmonella* spp. caused 4 % and *Campylobacter* spp. caused 2 %. No *Vibrio* species were isolated from the 437 samples.

Of the *E. coli* detected, the most frequent type was EAEC (43 %), followed by EPEC (29 %), ETEC (18 %) and EIEC (9 %). Of the 36 *Shigella* isolates, 36 % were *S. flexneri*, followed by *S. sonnei* (33 %) and *S. dysenteriae* (11 %). The *Salmonella* isolates represented two different serotypes, *S. typhi* (76 %) and *S. paratyphi* (24 %). All eight *Campylobacter* isolates were of *C. jejuni*.

Rotavirus was found in 22 % of the children with diarrhoea. The virus was most common in the 49–60 month age group.

Protozoic parasites were found in 16 % of the 437 children. *Giardia intestinalis* was found in 11 % of the children, followed by *Entamoeba histolytica* in 5 % of the children. All micro-organisms identified in the stool specimens are described in Table 2.

**Table 2.** Micro-organisms identified in stool samples from child diarrhoeal cases

Micro-organism	Number of cases (%)
Enteroaggregative *E. coli* (EAEC)	91 (21)
Enteropathogenic *E. coli* (EPEC)	61 (14)
Enterotoxigenic *E. coli* (ETEC)	39 (9)
Enteroinvasive *E. coli* (EIEC)	20 (4)
*Shigella sonnei*	12 (3)
*Shigella flexneri*	20 (4)
*Shigella dysenteriae*	4 (1)
*Salmonella typhi*	13 (2)
*Salmonella paratyphi* C	4 (1)
*Campylobacter jejuni*	8 (3)
*Giardia intestinalis*	47 (11)
*Entamoeba histolytica*	22 (5)
Rotavirus A	96 (22)
Total	437 (100)

Distribution of the diarrhoea among the 437 children according to age and gender in this study was as follows: 5 % were aged 0–6 months, 6 % were 7–24 months, 5 % were 25–36 months, 18 % were 37–48 months and 66 % were 49–60 months. Of the diarrhoea cases in the study, 240 (55 %) were boys and 197 (45 %) girls, giving a ratio male to female of 1 : 0.82 (Table 3). The rate of diarrhoea in the oldest group (49–60 months) of children and in the males was significantly higher (*P*<0.0001).

**Table 3.** Number of child diarrhoeal cases identified, subdivided according to child age and gender

Age group (months)	Males	Females	Total
0–6	7	13	20
7–24	9	19	28
25–36	8	15	23
37–48	23	56	79
49–60	193	94	287
Total	240	197	437
*P-*value (χ^2^-test)	<0.0001		

### Clinical features and seasonality

Of the 437 children with diarrhoea, 98 % had watery diarrhoea and 2 % had bloody diarrhoea. Fever was apparent in 53 % of the children, most commonly children with shigellosis and rotavirus. Vomiting was seen in 55 % of the children and dehydration in 60 % of the children. The viral infections tended to occur during winter, the bacterial infections during summer and autumn and parasitic infections were seen only during the rainy season.

### Antimicrobial sensitivity

All (211/211) of the *E. coli* isolates were sensitive to chloramphenicol, while 206 isolates of the 212 tested were sensitive to ceftazidime, 198 to ciprofloxacin, 200 to gentamicin, 160 to tetracycline, 150 to amikacin, 140 to nalidixic acid and only 100 to ampicillin (Table 4). Among the total *Shigella* species (*n* = 36), 30 were sensitive to chloramphenicol, 28 to ciprofloxacin and gentamicin, 24 to ceftazidime, 20 to tetracycline, 19 to amikacin, 18 to nalidixic acid and 18 to ampicillin (Table 5). Among the *Salmonella* spp. (*n* = 17), sensitivity to chloramphenicol, gentamicin and tetracycline was found in 17 of the isolates, while 15 isolates were sensitive to ceftazidime and ciprofloxacin and 11 isolates were sensitive to amikacin, nalidixic acid and ampicillin (Table 6). *Campylobacter* were sensitive to all antibiotics tested except for two isolates that were resistant to ampicillin.

**Table 4.** Antimicrobial sensitivity of *E. coli* isolates (*n* = 211)

Antibiotics	Sensitive (%)	Intermediate (%)	Resistant (%)
Amikacin	150 (71)	0	62 (29)
Ampicillin	100 (47)	12 (6)	100 (47)
Ceftazidime	206 (97)	0	6 (3)
Ciprofloxacin	198 (93)	2 (1)	12 (6)
Chloramphenicol	211 (100)	0	0
Gentamicin	200 (94)	3 (2)	9 (4)
Nalidixic acid	140 (66)	0	72 (34)
Tetracycline	160 (75)	2 (1)	50 (24)

**Table 5.** Antimicrobial sensitivity of *Shigella* spp. isolates (*n* = 36)

Antibiotics	Sensitive (%)	Intermediate (%)	Resistant (%)
Amikacin	19 (53)	3 (4)	14 (39)
Ampicillin	18 (50)	2 (6)	16 (44)
Ceftazidime	24 (67)	4 (11)	8 (22)
Ciprofloxacin	28 (78)	5 (14)	3 (8)
Chloramphenicol	30 (83)	2 (6)	4 (11)
Gentamicin	28 (78)	4 (11)	4 (11)
Nalidixic acid	19 (53)	5 (14)	12 (33)
Tetracycline	20 (56)	8 (22)	8 (22)

**Table 6.** Antimicrobial sensitivity of *Salmonella* isolates (*n* = 17)

Antibiotics	Sensitive (%)	Intermediate (%)	Resistant (%)
Amikacin	11 (64)	3 (18)	3 (18)
Ampicillin	11 (64)	2 (12)	4 (24)
Ceftazidime	15 (88)	2 (12)	0
Ciprofloxacin	15 (88)	0	2 (12)
Chloramphenicol	17 (100)	0	0
Gentamicin	17 (100)	0	0
Nalidixic acid	11 (64)	2 (12)	4 (24)
Tetracycline	17 (100)	0	0

## Discussion

In this study, we used a combination of conventional and molecular techniques to investigate the aetiological agent (bacterial, viral and parasitic pathogens) in stool samples from children with diarrhoea living in an urban area in Khartoum, Sudan. Of the 437 samples tested, 240 were from male children and 197 from females, giving a male to female ratio of 1.2 : 1. All children in the study were under 5 years of age. The largest number of samples was from the 49–60 month age group 287 (66 %), followed by the 37–48 month age group 79 (18 %). Among the total of 437 samples tested in the study, bacterial pathogens were detected in 272 (62 %) samples.

The rate of the diarrhoea was higher in male children, 240 (55 %), than in female children, 197 (45 %), as reported in many previous studies (Klein *et al.*, 2006; Moyo *et al.*, 2011; Shariff *et al.*, 2003; Sherchand *et al.*, 2009) and the ratio of male to female children affected by diarrhoeal disease was distinct from that in other studies, which found that male and female children were equally affected (Mashoto *et al.*, 2014). The majority of positive samples 366 (84 %) were found in children older than 2 years, particularly those aged 4–5 years as noted above. The prevalence of diarrhoea in children above 2 years was found to be significantly higher (*P*<0.01). Contaminated hands is one of the most common routes for transmission of food-borne infections, which might be one reason why diarrhoea was high in the age group 4–5 years, as poor hand washing practices in this age group are common.

The prevalence of diarrhoea caused by bacteria was significantly higher than that caused by viruses and parasitic infections, in contrast to results reported in other studies in different countries (Nitiema *et al.*, 2011).This is probably because in contrast to previous studies, we investigated the presence of different DEC subgroups and *Campylobacter,* and these findings increased the detection of bacteria as the causative agents of diarrhoea.

*E. coli* was the most frequently detected pathogen, in contrast to published findings for other developing countries (Aryal, 1996; Maharjan *et al.*, 2007; Mandomando *et al.*, 2007; Sherchand *et al.*, 2009). *E. coli* was detected in 48 % (211) of stool samples. EAEC was the most commonly detected type of *E. coli* in children in this study, present in 43 % (91) of cases, suggesting that it is the major cause of diarrhoea (Olesen *et al.*, 2005), whereas some previous studies concluded that it is not associated with diarrhoea (Bonkoungou *et al.*, 2012). However, those studies were conducted during the dry season, which may explain the differences. Therefore *E. coli* can be considered the most frequently isolated species of diarrhoeal disease in children in Khartoum.

*Shigella* species were detected in 36 (8 %) of the stool samples, with *S. flexneri* the most frequent isolate, 56 % (20/36), followed by *S. sonnei*, 33 % (12/36) and *S. dysenteriae,* 11 % (4/36). Other studies in Tanzania and in Jordan have reported similar results for shigellosis (Moyo *et al.*, 2011; Youssef *et al.*, 2000). *Salmonella* spp. were isolated from 4 % of the stool samples in this study. This percentage was similar to that obtained in others studies in East Africa, in Mozambique and Tanzania, where the prevalence was approximately 3 % (Mandomando *et al.*, 2007; Moyo *et al.*, 2011).

Among *Salmonella* spp., *S. typhi* was detected in 76 % (13/17) of cases and *S. paratyphi* C in 24 % (4/17). This is the first study to detect *C. jejuni* in Sudan. In fact, *Campylobacter* is not on the detection system in routine laboratory investigations on the aetiology of diarrhoea in Sudan.

Rotavirus was the second most common pathogen detected in our study, supporting the well-documented role of rotavirus in diarrhoea in children in the developing countries (Bonkoungou *et al.*, 2010; Nitiema *et al.*, 2011; O’Ryan *et al.*, 2005; Rodrigues *et al.*, 2007). The majority of rotavirus cases were detected in children aged over 2 years.

Intestinal parasites have always been an important public health problem in the developing countries, especially in tropical and subtropical areas (Easow *et al.*, 2005). The prevalence of intestinal parasites causing diarrhoea was found to be around 16 % (69/437) in this study, among which *G. intestinalis* represented 68 % (47/69) and *Entamoeba histolytica*, 32 % (22/69). These results confirmed previous findings reported for the Kathmandu Valley (Shrestha *et al.*, 2009; Thapa Magar *et al.*, 2011).

The presence of blood in 2 % (9/437) of samples may be due to *Entamoeba histolytica*. The low positive rate of intestinal parasites found in this study may be due to the source of drinking water used, the fact that public toilets were located at a distance from houses and the availability of a toilet in most houses.

The antimicrobial susceptibility tests showed that among the 211 *E. coli* isolates tested, 100 % of isolates were demonstrated to be sensitive to chloramphenicol, while 97 % (206/211) demonstrated sensitivity to ceftazidime, 94 % (200/211) to gentamicin, 93 % (198/211) to ciprofloxacin and 75 % (160/211) to tetracycline. Very few isolates were resistant to the different antibiotics but among these 34 % (62/211) were resistant to nalidixic acid and 47 % (100/211) were resistant to ampicillin. Previous studies found that ciprofloxacin was 100 % (8) effective against *E. coli* and tetracycline was effective in 29 % (101) of cases, but that 50 % (8) of these bacteria were resistant to ampicillin (Al-Gallas *et al.*, 2007; Kaminski *et al.*, 1994). Among the 36 isolates of the *Shigella* spp. tested here, 83 % (30/36) were sensitive to chloramphenicol and 78 % (28/36) were sensitive to ciprofloxacin and gentamicin, whereas 44 % (16/36) of the isolates were resistant to ampicillin and 39 % (14/36) to amikacin. Bodhidatta *et al.* (2002) found that 100 % (56/56) of bacteria isolates tested were sensitive to ciprofloxacin, unlike in our study. This may be due to empirical use of these antibiotics and development of resistance in Sudan. Chloramphenicol, gentamicin and tetracycline were the most effective antimicrobials for *Salmonella* spp. with 100 % (17/17) sensitivity, followed by 88 % (15/17) to ciprofloxacin and ceftazidime. This is in contrast to the findings of Mandomando *et al.* (2007) that 92 % (12) of *Salmonella* isolates were sensitive to chloramphenicol and 85 % (11) to tetracycline. That study also found that 62 % (8) of *Salmonella* isolates were resistant to ampicillin and 8 % (1) to nalidixic acid. This finding of much lower resistance to ampicillin in our study may be due to *Salmonella* not being a major problem in Khartoum, Sudan. In this study, chloramphenicol, gentamicin and third generation cephalosporin were the most effective antibiotics against the bacteria causing diarrhoea, while amikacin, ampicillin and nalidixic acid were the least effective. However, the use of chloramphenicol in children is not recommended (Mandomando *et al.*, 2007).

This study showed that the frequency of diarrhoea was higher in male children than in female children. Awareness about the prevention of the infectious diseases, improved hygiene and proper medication are needed to reduce the burden of the preventable infectious diseases among young children in Khartoum.
